# Heritability and prevalence of selected osteochondrosis lesions in yearling Thoroughbred horses

**DOI:** 10.1111/evj.12613

**Published:** 2016-09-04

**Authors:** J. Russell, O. Matika, T. Russell, R. J. M. Reardon

**Affiliations:** ^1^ Victorian Equine GroupBendigoVictoriaAustralia; ^2^ Roslin InstituteRoyal (Dick) School of Veterinary StudiesUniversity of EdinburghRoslinUK; ^3^ Department of SurgeryHospital for Large AnimalsRoyal (Dick) School of Veterinary StudiesUniversity of EdinburghRoslinUK

**Keywords:** horse, osteochondrosis, Thoroughbred, heritability

## Abstract

**Background:**

Osteochondrosis is considered multifactorial in origin, with factors such as nutrition, conformation, body size, trauma and genetics thought to contribute to its pathogenesis. Few studies have investigated the effects of genetic variability of osteochondrosis in Thoroughbreds.

**Objectives:**

To describe the prevalence and genetic variability of a subset of osteochondrosis lesions in a group of Thoroughbred yearlings.

**Study design:**

Retrospective cohort study.

**Methods:**

Radiographs of 1962 Thoroughbred yearlings were retrieved from clinical records obtained between 2005 and 2013. Pedigree information was obtained from the Australian Stud Book. Osteochondrosis lesions were documented in selected joints and estimates of heritability were obtained by fitting linear mixed models in ASREML software.

**Results:**

The overall prevalence of osteochondrosis was 23%. Osteochondrosis was identified in 10% of stifle joints, 6% of hock joints and 8% of fetlock joints. The heritability estimates ranged from 0 to 0.21. The largest estimates were 0.10, 0.14, 0.16 and 0.21 for lesions of the distal intermediate ridge of the tibia, dorso‐proximal proximal phalanx (P1), any stifle osteochondrosis, and lesions of the lateral trochlear ridge of the distal femur, respectively. Although calculated heritability estimates had high standard errors, meta‐analyses combining the present results with published estimates were significant at 0.10, 0.17, 0.15 and 0.20 for stifle, tarsal, fetlock and these joints combined, respectively. In addition, there was a permanent environment attributable to the dam effect.

**Main limitations:**

Inclusion criteria were based on radiographic findings in specific joints at a specific age range in Thoroughbreds.

**Conclusions:**

The present results indicate that only a proportion of osteochondrosis in Thoroughbreds is heritable. The permanent environment effects of the dam were observed to have effects on some categories of osteochondrosis.

## Introduction

Osteochondrosis is a common cause of lameness and joint effusion in the horse 1, 2 and may lead to reduced athletic performance 3, 4. Osteochondrosis is considered to be multifactorial in origin, with factors such as nutrition, conformation, body size and trauma thought to contribute to its pathogenesis 2, 5, 6, 7. Previous studies have reported a genetic control of osteochondrosis or other factors that lead to this condition 6, 8, 9.

Prevalence and heritability estimates for osteochondrosis vary among breeds 10, 11, 12, 13, which suggests the existence of genetic variability in disease susceptibility among breeds 14. Studies have shown osteochondrosis to be heritable in some predilection sites in Hanoverian Warmblood horses 10 and Standardbred trotters 11. A study in Thoroughbreds examining quantitative trait loci for osteochondrosis, in conjunction with clinically diagnosed (radiographs and arthroscopy) cases, was unable to identify significant heritability, which may have been related to a lack of power 14. Another study of Thoroughbreds, conducted using data derived from radiographic reports, described a significant genetic component to osteochondrosis 15.

The Thoroughbred population can be considered closed, as most of the pedigree can be traced back to three foundation sires in the 17th century, 78% of alleles are derived from 30 foundation mares, and 95% of paternal lineages can be attributed to one stallion 16. Breeding practices also contribute to the uniqueness of the Thoroughbred population as artificial insemination is not permitted in this breed 17.

Osteochondrosis is a dynamic disorder in which lesions develop gradually in the first few months of life, most of which then heal spontaneously 18. Normal and abnormal radiographic appearances are reportedly permanent in the tarsocrural joint after 5 months of age and in the femoropatellar joint after 8 months of age 18.

In the absence of clinically apparent disease in young Thoroughbreds, the diagnosis of osteochondrosis is commonly made at the time that pre‐sale radiographic data are acquired 4, 8. The advent of sales radiograph repositories has provided opportunities to evaluate radiographic findings in multiple joints of large numbers of young Thoroughbred horses 15, 19, 20. Although the information gained from repositories provides a useful overview, it is subject to bias as animals presented for sale are unlikely to have significant osteochondrosis lesions, or may have been submitted to surgery so that they appear radiographically normal.

The aim of this study was to describe the prevalence and genetic variability of a subset of osteochondrosis lesions in Thoroughbred yearlings in which documented surgical history and screening radiographs were available irrespective of whether they were subsequently presented for sale.

## Materials and methods

### Animals

Radiographs of 1962 yearlings imaged by an equine practice in Australia, prior to sales occurring between 1 November 2005 and 31 July 2013, were retrieved from clinical records. Details of each horse were obtained, including year of birth, sex and farm of origin. Farm of origin was defined as the property at which the foal had been born, raised and radiographed before being sent to any other farm to be prepared for sale.

Veterinary services were provided by the same practice for all animals from birth. Full medical histories were reviewed to identify horses that had received surgery to remove lesions at an earlier age. Feeding, housing and exercise levels varied among the animals. Animals that did not have a full history were excluded from the study.

Pedigree information for each horse was collected from the Australian Stud Book. Yearlings for which pedigree information to at least grand dam and grand sire levels was not available were excluded from the study.

### Radiographs

Each yearling had undergone a standardised radiographic screening consisting of 34 views as a routine procedure prior to potential sale. The details of the radiographic technique used in this study have been reported previously 19 and are summarised in Supplementary Item 1 (online). Radiographs were stored and reviewed using DICOM files and OsiriX software 21. Yearlings were excluded from analysis if a complete radiographic set was not available, if any radiograph was judged to be of non‐diagnostic quality, and if the animal could not be identified from the radiograph labelling. All radiographs were examined by two veterinary surgeons experienced in equine orthopaedics and radiology. Each examiner was blinded to the assessment of the other clinician and to the clinical history of each horse. Cases in which discrepancies in radiographic diagnosis occurred were reviewed by both examiners and a third opinion was sought from another registered specialist in equine surgery, after which a consensus decision was agreed. The radiographs were examined for the presence or absence of osteochondrosis lesions in the medial and lateral femorotibial and femoropatellar joints (stifle), and the tarsal, metacarpo‐ and metatarsophalangeal (fetlock) joints. The diagnosis of osteochondrosis was based on criteria defined by previous reports 11, 12, 13, 15, which included radiographic evidence of flattening or indentation of the articular surface and subchondral bone, or irregularly shaped radiolucent zones with surrounding sclerosis, with or without osteochondral fragments. The results of the examination were recorded as the presence or absence (0/1) of the conditions being investigated. The trait osteochondrosis included all radiographic findings consistent with osteochondrosis at one of the predilection sites, including osteochondrosis dissecans 15, but excluding palmar/plantar proximal phalangeal osteochondral fragments, which are not considered to be osteochondrosis lesions 22.

### Selection of osteochondrosis lesions for further evaluation

After radiograph evaluations, osteochondrosis lesions were chosen for variance component analysis based on frequency of occurrence, potential clinical relevance and whether they had been previously investigated. The following lesions were chosen for further analyses: any osteochondrosis; subchondral bone cysts in the medial femoral condyle (stifle cyst); lesions of the lateral trochlear ridge of the distal femur; any stifle osteochondrosis; lesions of the distal intermediate ridge of the tibia; lesions of the lateral trochlear ridge of the talus; any tarsal osteochondrosis; dorsoproximal P1 fragments (DP‐P1) (fore‐ and hindlimbs); proximal third metacarpal/metatarsal sagittal ridge lesions; and any fetlock osteochondrosis. Of 1962 yearlings initially available, 87 were excluded because radiographic sets were incomplete, 36 were excluded for radiographs of non‐diagnostic quality, 92 could not be identified in the stud book because the radiograph had been incorrectly labelled, and 76 were excluded for lack of surgical history. The remaining 1671 yearlings (1003 colts, 668 fillies) from 42 farms were used in all subsequent analyses.

### Pedigree

The available pedigree comprised 5249 animals over five generations with 677 sires mated to 2726 dams. Summaries of pedigree and observed data structure are presented in Supplementary Item 2 (online).

### Data analyses

Interobserver agreement between the two primary observers was assessed using correlation analyses in Stata Version 12.0^1^.

Estimates of heritability were obtained using variance component analyses in ASREML software^2^ ^23^. Selected osteochondrosis lesions were analysed with the phenotypes treated either as continuous or binary (0/1) traits. The binary traits were analysed using linear mixed models and generalised linear models with a logit transformation fitting a logit link function. When a binary trait is considered, estimation of its heritability using generalised linear mixed models (i.e. models using a link function to make the residuals normally distributed) is more reliable when using a sire model than when considering animal as a random effect 23. The non‐genetic effects fitted included sex (male and female) and year in which the yearling was presented for examination. The random genetic effects explored in the model included those attributable to the animal and sire. Other non‐genetic random effects fitted were those for permanent environmental effects attributable to the mare and the farm. The permanent environmental effects model showed within‐dam environmental variation across foalings. The models fitted were as follows:

(1) $$y=Xb+Zu+Zpe+{\text{Wv}}_{farm}+e$$

(2) $$y=X\beta +{\text{Zv}}_{sire}+Zpe+{\text{Wv}}_{farm}+e$$

where *y* is a vector of observations on the specific trait; *b* is vectors of non‐genetic fixed effects; *u* (additive), v_*sire*_, *pe* and v_*farm*_ are vectors of random effects for direct additive genetic effects, sire, and permanent environment effects attributable to the mare and farm, respectively; X is the corresponding incidence matrix relating the observations to *b*, Z is the corresponding incidence matrix relating *u* or *sire*,* pe* to y, W is the corresponding incidence matrix relating *farm* to y, and *e* is the residual. The normal distributions assumed for the random effects were as follows: animal ~N (0, Aσ^2^_*a*_), ~N (0, Aσ^2^_*sire*_), pe ~ N (0, Iσ^2^_*pe*_), ~N (0, Iσ^2^_*farm*_), e ~ N (0, Iσ^2^_*e*_), where A is the numerator relationship matrix, and I represents identity matrices of the order equal to the number of farms and records. The relationship matrix was constructed using pedigree records of 5249 horses. The narrow‐sense heritabilities (h^2^) were estimated as follows: animal model: h^2^ = σ^2^_*additive*_/(σ^2^_*additive*_ + σ^2^_*pe*_ + σ^2^_*farm*_σ + σ^2^_*residual*_), sire model h^2^ = 4σ^2^_*sire*_/(σ^2^_*sire*_ + σ^2^_*pe*_ + σ^2^_*farm*_ + σ^2^_*residual*_). Estimates of underlying heritability were obtained from mixed linear model estimates and then transformed following Dempster and Lerner 24.

A meta‐analysis was performed to obtain estimates of mean heritabilities from published values across breeds, together with standard errors to account for sampling and population variation. The analysis was performed only where published heritability estimates and standard errors were available, for osteochondrosis lesions in the stifle, tarsus and fetlocks, individually or combined, using methods described earlier 25.

## Results

### Prevalence

The prevalence of horses with at least one osteochondrosis lesion was 23%. Osteochondrosis lesions in stifle joints were identified in 10% of horses, with the most common site being the lateral trochlear ridge of the distal femur (6% of horses). Osteochondrosis lesions in the tarsus were observed in 6% of horses, with the most common site being the distal intermediate ridge of the tibia (4% of horses). Osteochondrosis of the fetlock joints was identified in 8% of horses, the most common site being DP‐P1, observed in 4% of horses. The summary of frequencies of different lesions subdivided by joints, alongside comparisons with published figures, is shown in Table 1.

**Table 1:** Frequencies of lesions identified in 1671 horses in this study and comparisons with published data

Lesion site	*n* (%)	Percentage range from other studies of TBs	Percentage range from studies of non‐TBs	References: TB (non‐TB)
Any osteochondrosis	389 (23.3%)	20.5%	16.6%	15 (32)
Any stifle osteochondrosis	166 (9.9%)	6.2–10.2%	11.5–16.6%	20,26,27 (31,32)
Medial femoral condyle cyst	63 (3.8%)	0–5.6%		20,26,27
Bilateral medial femoral condyle cyst	13 (0.8%)			
Lateral trochlear ridge of femur	104 (6.2%)	4.0–5.7%		26,27
Bilateral lateral trochlear ridge	44 (2.6%)			
Any tarsal osteochondrosis	102 (6.1%)	6.5–10.5%	9.2–16.0%	20,26,27 (32,33)
Distal intermediate ridge of tibia	60 (3.6%)	3.0–4.4%		26,27
Bilateral distal intermediate ridge of tibia	9 (0.5%)			
Lateral trochlear ridge of talus	39 (2.3%)	1.4–4.0%		20,26
Bilateral lateral trochlear ridge of talus	8 (0.5%)			
Medial malleolus of distal tibia	3 (0.2%)			
Both lateral trochlear ridge and distal intermediate ridge of tibia	8 (0.5%)			
Any metacarpo/tarsophalangeal joint osteochondrosis	134 (8.0%)	14.6–15.4%	11.8–23.5%	26,27 (33,34)
Any distal third metacarpal/metatarsal cyst	16 (1.0%)	2.0–6.0%		20,26,27
Distal third metacarpal cyst	10 (0.6%)			
Distal third metatarsal cyst	6 (0.4%)			
Any dorso‐proximal P1 (DP‐P1) osteochondral fragment	62 (3.7%)	3.0–4.9%		20,26,27
Forelimb DP‐P1 fragment	24 (1.4%)			
Hindlimb DP‐P1 fragment	38 (2.3%)			
Bilateral forelimb DP‐P1 fragment	3 (0.2%)			
Bilateral hindlimb DP‐P1 fragment	5 (0.3%)			
Any P1 cyst	14 (0.8%)			
Forelimb P1 cyst	5 (0.3%)			
Hindlimb P1 cyst	11 (0.7%)			
Bilateral hindlimb P1 cyst	0			
Any distal third metacarpal/tarsal proximal sagittal ridge fragment	37 (2.2%)			
Third metacarpal proximal sagittal ridge fragment	22 (1.3%)			
Bilateral third metacarpal proximal sagittal ridge fragment	7 (0.4%)			
Third metatarsal proximal sagittal ridge lesion	23 (1.4%)			
Total number of osteochondrosis sites	505 (30.2%)			

TB, Thoroughbred; non‐TB, breed other than Thoroughbred.

### Observer agreement

Results of kappa analysis identified high levels of interobserver agreement ranging from 99.9% to 100% for the osteochondrosis lesions examined.

### Estimates of heritability

The estimates of genetic variances and heritability from the sire logit model are shown in Table 2. Other heritability estimate results from the animal and sire models when treated as binary or continuous traits are presented in Supplementary Items 3–5 (online) for comparison. The heritability estimates ranged from 0 to 0.21 but had large standard errors, and none were significantly different from zero. The largest estimates were 0.10, 0.14, 0.16 and 0.21 for lesions of the distal intermediate ridge of the tibia, DP‐P1, any stifle osteochondrosis and lesions of the distal lateral trochlear ridge of the distal femur, respectively (Table 2). These estimates were similar to some of the underlying heritability estimates calculated as a continuous trait using the animal model (Supplementary Item 3).

**Table 2:** Summary of estimates of variance and heritability obtained from a sire model using the logit link function for some osteochondrosis lesions

Trait (osteochondrosis lesion site)	σ^2^_*sire*_	σ^2^_*phen*_	s.e.	h^2^	s.e.	PE	s.e.
Any osteochondrosis	0.076	3.649	0.158	0.08	0.06	0.07	0.04
Stifle cyst	0.000	3.468	0.656	0.00	0.00	0.05	0.18
Lateral trochlear ridge of distal femur	0.212	4.092	0.395	0.21	0.17	0.13	0.08
Any stifle osteochondrosis	0.155	3.884	0.281	0.16	0.12	0.11	0.06
Distal intermediate ridge of tibia	0.092	3.532	0.627	0.10	0.19	0.04	0.17
Lateral trochlear ridge of talus	0.000	4.037	0.758	0.00	0.00	0.18	0.15
Any tarsal osteochondrosis	0.000	3.527	0.392	0.00	0.00	0.06	0.10
Dorsoproximal P1	0.126	3.559	0.657	0.14	0.24	0.00	0.18
Proximal sagittal ridge of Mc/MtIII	0.053	3.813	0.922	0.05	0.23	0.12	0.21
Any fetlock osteochondrosis	0.013	3.412	0.348	0.02	0.10	0.14	0.10

σ^2^_*sire*_, variation attributable to the sire; σ^2^_*phen*_, variation attributable to the phenotype; s.e., standard error of value to the left; h^2^, heritability estimate; PE, proportion of variation attributable to the permanent environment due to the dam.

The permanent environment attributable to the dam was not observed to be important in considerations of the sire logit model. However, in considerations of the linear models, significant estimates were observed for any osteochondrosis, any stifle osteochondrosis and lesions of the lateral trochlear ridge of the distal femur (Supplementary Items 3 and 4).

### Meta‐analysis

The heritability estimates for osteochondrosis obtained using the meta‐analysis were low, ranging from 0.10 to 0.20 for stifle osteochondrosis and for combined stifle, tarsus and fetlock osteochondrosis, respectively. A detailed summary of the meta‐analysis results and the published estimates used is given in Table 3.

**Table 3:** Summary estimates of heritability calculated using a meta‐analysis of heritability estimates for osteochondrosis in different joints in published data and the current study

Sample description	Method used	h^2^ (s.e.)	Reference
Stifle osteochondrosis	Meta‐analysis	0.10 (0.04)	
1671 TBs	SM	0.16 (0.12)	Current study
1004 TBs	LAM II	0.10 (0.10)	Castle 15
7396 Hanoverian WBs	LAM I	0.21 (0.04)	Hilla and Distl 10
1734 Swedish WBs	LAM I	0.03 (0.04)	Jonsson *et al*. 12
811 Dutch WBs	LAM II	0.05 (0.05)	Grevenhof *et al*. 36
Tarsal osteochondrosis	Meta‐analysis	0.17 (0.06)	
1671 TBs	SM	0.00 (0.00)	Current study
1004 TBs	LAM II	0.10 (0.11)	Castle 15
1217 STBs	SM III	0.29 (0.15)	Lykkjen *et al*. 11
7396 Hanoverian WBs	LAM I	0.35 (0.04)	Hilla and Distl 10
1785 Swedish WBs	LAM I	0.08 (0.04)	Jonsson *et al*. 12
793 Swedish STBs	LSM I	0.24 (0.19)	Philipsson *et al*. 37
167 SGCs	LAM I	0.02 (0.04)	Wittwer *et al*. 38
811 Dutch WBs	LAM II	0.36 (0.11)	Grevenhof *et al*. 36
Fetlock osteochondrosis	Meta‐analysis	0.15 (0.03)	
1671 TBs	SM	0.02 (0.10)	Current study
7396 Hanoverian WBs	LAM I	0.17 (0.03)	Hilla and Distl 10
811 Dutch WBs	LAM II	0.14 (0.08)	Grevenhof *et al*. 36
167 SGCs	LAM I	0.16 (0.16)	Wittwer *et al*. 38
Fetlock/tarsal/stifle osteochondrosis^a^	Meta‐analysis	0.20 (0.08)	
1671 TBs	SM	0.08 (0.06)	Current study
350 WBs	LAM I	0.14 (0.23)	Pieramati *et al*. 31
811 Dutch WBs	LAM II	0.23 (0.09)	Grevenhof *et al*. 36
3199 Swedish WBs	LAM I	0.05 (0.03)	Jonsson *et al*. 12

a Fetlock osteochondrosis includes fore‐ and hindlimbs. h^2^, heritability estimate; TBs, Thoroughbreds; WBs, Warmbloods; STBs, Standardbreds; SGCs, South German Coldbloods; LAM, linear animal model; SM, sire model; LSM, linear sire model; I, heritability estimates using a linear model then transformed following Dempster and Lerner 24; II, heritability estimates after prior transformation to a continuous liability scale; III, heritability estimates from a sire model with prohibit transformation.

## Discussion

The current study benefited from its access to a large number of radiographs obtained prior to potential sales. This offered an opportunity to study osteochondrosis lesions in a horse population intended to be trained for racing but before being subject to selection as in other published studies 26, 27. Additionally, access to the full medical and surgical histories reduced the likelihood that animals in which an osteochondrosis lesion(s) had been removed at an earlier age would be included.

The diagnostic criteria for osteochondrosis lesions, which were based on published methods 11, 12, 13, 15, may have resulted in the inclusion of some mild cases of osteochondrosis; however, other more definitive changes, such as osseous fragments and subchondral cysts, were also reported. This approach was chosen in an attempt to maximise the usefulness of the information derived from radiographs. To maximise power and simplify analysis, the radiographic severity of osteochondrosis lesions was not included in the analyses.

Radiographic evidence of osteochondrosis can change in growing animals and most lesions heal over time. Those lesions that remain after the animal has reached 12 months of age are likely to be permanent 18, 28. Animals in this study were aged 14–21 months and thus it is possible that more lesions that subsequently healed may have been present at a younger age. As a result the heritability estimates presented should perhaps be defined as applicable to ‘permanent osteochondrosis lesions’ rather than to ‘any osteochondrosis lesion’. This is a limitation of this study and of all heritability estimates of osteochondrosis in horses reported to date. Further, the inclusion of only certain joints is likely to have resulted in a slight underestimation of the prevalence of osteochondrosis, as the condition can occur (albeit infrequently) in other articulations in the horse, such as those of the cervical vertebrae 29. Osseous cyst‐like lesions may also occur in many locations, such as the distal radius, proximal tibia and bones of the phalanx, that may not be detected in the standard pre‐sale radiographic set. Additionally, subclinical and radiographically invisible osteochondrosis lesions have been documented 2, 29, 30, but the fact that these would not have been identified in this study may have led to further underestimation. The radiographs in the current study were not interpreted by recognised specialists in diagnostic imaging, which may be considered a limitation. However, the two equine veterinary surgeons who participated in the study had extensive experience in interpreting these types of radiographic study and any cases for which discrepancies arose in interpretations were reviewed by a third equine veterinarian with more than 35 years of experience in interpreting Thoroughbred sales radiographs. Correlation analysis identified excellent agreement between observers.

The overall prevalence of osteochondrosis (23%) was similar to that of 20.5% reported in another study of Thoroughbreds in Australia conducted by Castle in 2012 15. Castle collected information from veterinary reports on radiographic studies performed in Thoroughbred yearlings 15. Each report was written by one of 16 veterinarians from various private equine practices. The participation of only two veterinarians in the current study reduced the likelihood of interobserver discrepancies. The prevalences of some of the specific osteochondrosis lesions identified in the stifle and tarsus were similar to those in other reports of Thoroughbreds: stifle, 10% (reported prevalences: 6.2–10.2% 20, 26, 27), and tarsal, 7.2% (reported prevalences: 6.5–10.5% 20, 26, 27). However, they were slightly lower than those reported in other breeds, in which reported prevalences are: stifle, 11.5–16.6% 31, 32, and tarsal, 9.2–30.0% 32, 33. The prevalence of fetlock osteochondrosis (8%) was lower than those reported in both Thoroughbreds (14.6–15.4%) 25, 26 and other breeds (11.8–23.5%) 33, 34.

The direct comparison of prevalences may not be appropriate because sampling strategies, categorisation schemes, radiographic examinations and the rigour with which radiographs were analysed for inclusion in the study may differ 20.

Only limited genetic variation was identified in the osteochondrosis lesions under study. These low estimates were associated with relatively high standard errors, which suggests that non‐genetic factors had a substantial influence on most osteochondrosis lesions. Because of the high standard errors, to confirm the validity of the models, estimates of the heritabilities and the permanent environment attributable to the dam were plotted across a range of log likelihoods for the outcome ‘any osteochondrosis’, which indicated that they were not adversely affected by the data structure (Supplementary Item 6 [online]) and that the estimates are likely to be significantly different from zero. The highest heritability estimates were identified for osteochondrosis lesions on the lateral trochlear ridge of the distal femur (0.21), anywhere in the stifle (0.16), and on the distal intermediate ridge of the tibia (0.10), which were generally higher than those (0.22, 0.10 and 0.00, respectively) reported in another study of Australian Thoroughbreds 15. Similarly, another study of Thoroughbreds reported non‐significant low estimates of heritability for osteochondrosis 14.

Studies of other horse breeds (predominantly Warmbloods), some of which included samples smaller than that used in this study, have reported higher levels of heritability for some of the osteochondrosis lesions examined. Results of the meta‐analyses suggest a genetic component to osteochondrosis, with heritabilities significantly different from zero. If the results of the meta‐analyses are applicable irrespective of horse breed, it should be possible to use selective breeding strategies to reduce the incidence of osteochondrosis in equine populations. A study of Maremanno horses, having estimated a heritability of 0.14, demonstrated the potential to reduce the incidence of osteochondrosis dissecans from current levels of 17% to around 2% by selective breeding of males and females over five generations 31. Further examination of Thoroughbred populations would be required to confirm or dispute significant heritability before this approach can be attempted.

Although the estimates of heritability for the osteochondrosis lesions examined in this study were low and had large standard errors, three categories of osteochondrosis (any osteochondrosis lesion, any stifle osteochondrosis, and lesions of the lateral trochlear ridge of the distal femur) were significantly associated with the permanent environment attributable to the dam in the linear models. This may suggest that breeding and farm management practices (e.g. feeding), or other factors, such as parity or age of mares, may be related to the likelihood of osteochondrosis and could potentially be used to alter the prevalence of osteochondrosis lesions in the Thoroughbred. There would be potential significant benefits for the breeding industry if these practices could be identified. A recent study in Warmblood horses identified a significant relationship between the development of osteochondrosis and maternal nutrition during gestation, with mares that were fed concentrates during gestation being more likely to have foals that developed osteochondrosis 35. Further investigation of the relationship between the permanent environment attributable to the mare, including management regimens practised, and detailed dietary histories during gestation, and the likelihood of osteochondrosis in the Thoroughbred may be warranted.

## Conclusions

The overall prevalence of osteochondrosis (23%) in yearling Thoroughbreds was considerable and comparable with those in other breeds. Although the present estimates are low and are associated with high standard errors, those from the meta‐analyses, which included the current study, indicate that regardless of horse breed, a significant, relatively modest genetic component of osteochondrosis exists (0.10–0.20). These findings warrant future studies that ideally should include radiographic studies monitoring for the presence of osteochondrosis in the first 8 months of life, the age of the mare and any relevant information on the farms and management regimens practised.

## Authors’ declaration of interests

No competing interests have been declared.

## Ethical animal research

This was a retrospective study of clinical records and data obtained from public sources and hence did not require research ethics committee oversight. Farm managers gave consent for their horses to be included in this study.

## Source of funding

O. Matika was supported by the Biotechnology and Biological Sciences Research Council (BBSRC) Institute Strategic Project (grant BBS/E/D/20231760).

## Authorship

J. Russell conducted most of the work in this study. He performed the radiographic analysis, all data input and the literature search, and prepared most of the manuscript. R. Reardon contributed to data analysis and interpretation, wrote the results section and much of the discussion, and brought the parties together to allow this manuscript to be produced. O. Makita performed most of the complex statistical analysis and contributed to the preparation of the manuscript. T. Russell conceived and designed the study, provided all cases, performed all radiographic interpretation and co‐ordinated the progress of the work. All authors approved the final version of the manuscript for publication.

## Supporting information


**Supplementary Item 1:** Radiographic views.

**Supplementary Item 2:** Pedigree data structure.

**Supplementary Item 3:** Estimates of variance and heritability obtained from an animal model for some osteochondrosis lesions.

**Supplementary Item 4:** Estimates of variance and heritability obtained from a sire model for some osteochondrosis lesions.

**Supplementary Item 5:** Estimates of variance and heritability obtained from an animal model using the logit function for some osteochondrosis lesions.

**Supplementary Item 6:** A three dimensional plot of estimates of additive and permanent environmental effects due to the mare, for the trait “any osteochondrosis” analysed fitting a sire model. Colour scale is a log‐likelihood heat map.

## Manufacturers' addresses

1 StataCorp LP, College Station, Texas, USA.

2 VSN International Ltd, Hemel Hempstead, Hertfordshire, UK.
