# Supplementary material for: Heritability and prevalence of selected osteochondrosis lesions in yearling Thoroughbred horses
Source: Equine Vet J. 2016 Sep 4;49(3):282–7. doi: 10.1111/evj.12613 (PMC5412687; doi:10.1111/evj.12613)
Supplement: Supplementary file 1 — Supplementary Item 1: Radiographic views. [file EVJ-49-282-s001.pdf]

Equine Veterinary Journal

Supplementary Item 1: Complete list of 34 radiographic views. Each view is repeated for left and right limbs.

**Fore feet**	Lateromedial (LM)
**Fore fetlocks**	Flexed lateromedial (flexed LM)
	Dorso 45-55 degree lateral-palmaromedial elevated 5-10 degree oblique (DLPaMO)
	Dorso 45-55 degree medial-palmarolateral elevated 5-10 degree ooblique (DMPaLO)
	Dorsopalmar elevated 20 degree (DPa)
**Carpi**	Flexed lateromedial (Flexed LM)
	Dorso 50-60 degree lateral-palmaromedial oblique (DLPMO)
	Dorso 70 degree medial-palmarolateral oblique (DMPLO)
**Hind fetlocks**	Lateromedial (LM)
	Dorso 45-55 degree lateral-palmaromedial 15-20 degree elevated oblique (DLPlMO)
	Dorso 45-55 degree medial-palmarolateral 15-20 degree elevated oblique (DMPlLO)
	Dorsopalmar 30 degree elevated (DPl)
**Tarsi**	Lateromedial (LM)
	Dorso 50-60 degree medial plantarolateral oblique (DMPlLO)
	Dorso 10-20 degree lateral palmaro medial oblique (DPl)
**Stifles**	Lateromedial (LM)
	Caudocranial (CdCr)
