# Supplementary material for: Heritability and prevalence of selected osteochondrosis lesions in yearling Thoroughbred horses
Source: Equine Vet J. 2016 Sep 4;49(3):282–7. doi: 10.1111/evj.12613 (PMC5412687; doi:10.1111/evj.12613)
Supplement: Supplementary file 2 — Supplementary Item 2: Pedigree data structure. [file EVJ-49-282-s002.pdf]

Equine Veterinary Journal

Supplementary Item 2: Summary statistics on pedigree data structure

Description	Number of animals
Total Pedigree	5,249
Sires	677
Sires of Sire	111
Dams of Sire	231
Dams	2,726
Sires of Dam	487
Dams of Dam	1,239
