# Supplementary material for: Heritability and prevalence of selected osteochondrosis lesions in yearling Thoroughbred horses
Source: Equine Vet J. 2016 Sep 4;49(3):282–7. doi: 10.1111/evj.12613 (PMC5412687; doi:10.1111/evj.12613)
Supplement: Supplementary file 4 — Supplementary Item 4: Estimates of variance and heritability obtained from a sire model for some osteochondrosis lesions. [file EVJ-49-282-s004.pdf]

Equine Veterinary Journal

Supplementary Item 4: Summary of estimates of variance and heritability obtained from a sire model for some osteochondrosis lesions.

Trait (osteochondrosis lesion site)	s^2_{Sire}	s^2_{Phen}	SE	h^2	SE	$h^2_{Underlying}$	PE	SE
Any osteochondrosis	0.002	0.178	0.006	0.05	0.04	***0.11***	***0.11***	0.04
Stifle cyst	0.000	0.036	0.001	0.00	0.00	0.00	0.01	0.04
Lateral trochlear ridge of distal femur	0.001	0.058	0.002	0.05	0.04	0.28	***0.15***	0.04
Any stifle osteochondrosis	0.001	0.089	0.003	0.06	0.04	***0.23***	***0.13***	0.04
Distal intermediate ridge of the tibia	0.000	0.035	0.001	0.01	0.03	***0.08***	0.01	0.03
Lateral trochlear ridge of talus	0.000	0.023	0.001	0.00	0.00	0.00	0.05	0.03
Any tarsal osteochondrosis	0.000	0.057	0.002	0.00	0.00	0.00	0.02	0.03
Dorso-proximal P1	0.000	0.036	0.001	0.02	0.03	***0.09***	0.00	0.04
Proximal sagittal ridge of MC/MT3	0.000	0.038	0.005	0.01	0.01	***0.04***	0.00	0.00
Any fetlock osteochondrosis	0.000	0.074	0.003	0.00	0.02	0.01	0.00	0.04

Key: s^2_{Sire} = variation attributable to the sire; s^2_{Phen} = variation attributable to the phenotype; SE = standard error of the column to the left; h^2 = heritability estimate; $h^2_{underlying}$ = underlying heritability estimate; PE = proportion of variation attributable to the permanent environment due to the dam. Significant estimates in bold italics.
