# Supplementary material for: Heritability and prevalence of selected osteochondrosis lesions in yearling Thoroughbred horses
Source: Equine Vet J. 2016 Sep 4;49(3):282–7. doi: 10.1111/evj.12613 (PMC5412687; doi:10.1111/evj.12613)
Supplement: Supplementary file 5 — Supplementary Item 5: Estimates of variance and heritability obtained from an animal model using the logit function for some osteochondrosis lesions. [file EVJ-49-282-s005.pdf]

Equine Veterinary Journal

Supplementary Item 5: Summary of estimates of variance and heritability obtained from an animal model using the logit function for some osteochondrosis lesions.

Trait (osteochondrosis lesion site)	s^2_{Anim}	s^2_{PE}	s^2_{Phen}	SE	h^2	SE	PE	SE
Any osteochondrosis	0.210	0.173	3.681	0.165	0.06	0.04	0.05	0.05
Stifle cyst	0.000	0.168	3.468	0.656	0.00	0.00	0.05	0.18
Lateral trochlear ridge of distal femur	0.413	0.339	4.109	0.403	0.10	0.11	0.08	0.12
Any stifle osteochondrosis	0.321	0.287	3.908	0.288	0.08	0.08	0.07	0.08
Distal intermediate ridge of the tibia	0.418	0.000	3.718	0.513	0.11	0.11	0.00	0.00
Lateral trochlear ridge of talus	0.000	0.737	4.037	0.758	0.00	0.00	0.18	0.15
Any tarsal osteochondrosis	0.090	0.170	3.561	0.414	0.03	0.10	0.05	0.13
Dorso-proximal P1	0.103	0.015	3.520	0.681	0.03	0.18	0.00	0.22
Proximal sagittal ridge of MC/MT3	0.497	0.135	3.930	0.950	0.13	0.23	0.03	0.28
Any fetlock osteochondrosis	0.078	0.000	3.430	0.283	0.02	0.08	0.00	0.00

Key: s^2_{Anim} = variation attributable to the animal; s^2_{PE} = variation attributable to permanent environment due to the dam; s^2_{Phen} = variation attributable to the phenotype; SE = standard error of the column to the left; h^2 = heritability estimate; PE = proportion of variation attributable to the permanent environment due to the dam. Significant estimates in bold italics.
