# Supplementary material for: Heritability and prevalence of selected osteochondrosis lesions in yearling Thoroughbred horses
Source: Equine Vet J. 2016 Sep 4;49(3):282–7. doi: 10.1111/evj.12613 (PMC5412687; doi:10.1111/evj.12613)
Supplement: Supplementary file 6 — Supplementary Item 6: A three dimensional plot of estimates of additive and permanent environmental effects due to the mare, for the trait “any osteochondrosis” analysed fitting a sire model. Colour scale is a log‐likelihood heat map. [file EVJ-49-282-s006.docx]

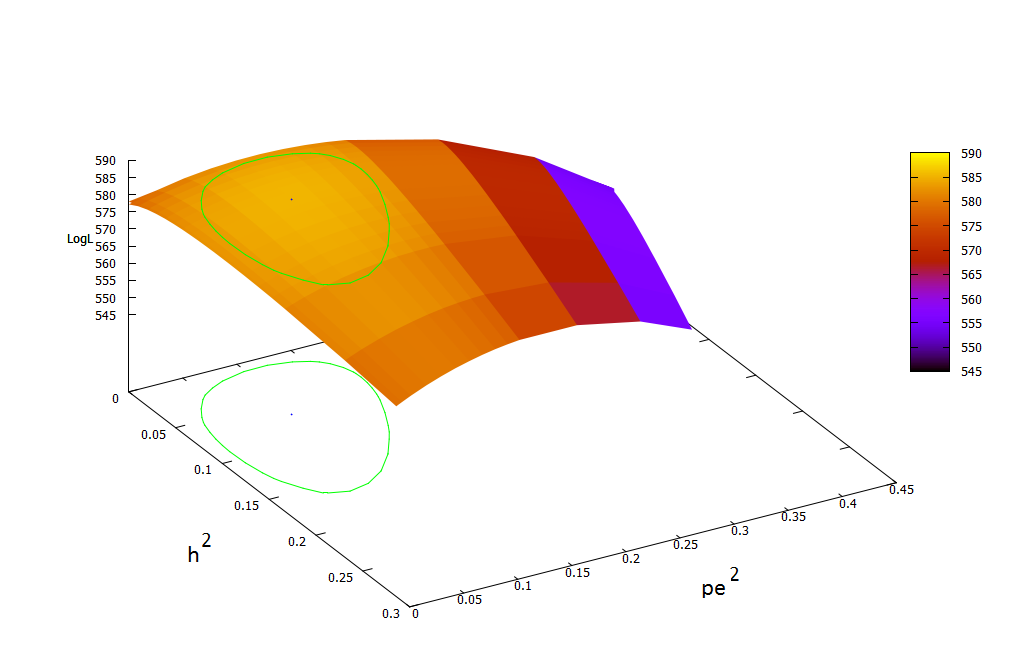


Supplementary item 6: A three dimensional plot of estimates of additive and permanent environmental effects due to the mare, for the trait “any osteochondrosis” analysed fitting a sire model. Colour scale is a log-likelihood heat map. X-axis represents the proportion of estimate attributable to permanent environment of the mare (pe^2^); Y-axis represents the heritability estimates for the additive genetics (h^2^); Z-axis represents the log-likelihood (LogL). The Black dot represents the highest log-likelihood value with the Green line representing the confidence interval allowing for a drop of 2 units from the maximum LogL value.
